# Lifestyle behaviors and mental health of health professional students during COVID-19, as measured by the CDC’s BRFSS, for the HOLISTIC cohort study

**DOI:** 10.1371/journal.pmen.0000302

**Published:** 2025-04-21

**Authors:** Atithi Patel, Jun Lu, Jyotsna Bitra, Sunil Dommaraju, Daniel Loizzo, Brenda Guillen, Niamh Kane, Danielle Westnedge, Jessica Lopez Guzman, Nancy Giang, Isabella Hartnett, Mary T. Keehn, Rashid Ahmed, Jerry A. Krishnan, Konadu Fokuo

**Affiliations:** 1 College of Medicine, University of Illinois, Chicago, Illinois, United States of America; 2 School of Public Health, University of Illinois, Chicago, Illinois, United States of America; 3 College of Applied Health Sciences, University of Illinois, Chicago, Illinois, United States of America; 4 Jane Addams College of Social Work, University of Illinois, Illinois, United States of America; 5 College of Pharmacy, University of Illinois, Chicago, Illinois, United States of America; 6 College of Nursing, University of Illinois, Chicago, Illinois, United States of America; 7 College of Community Health, Montclair State University, Montclair, New Jersey, United States of America; 8 Office of Population Health Sciences, University of Illinois, Chicago, Illinois, United States of America; 9 Department of Psychiatry, University of Illinois, Chicago, Illinois, United States of America; FMUSP: Universidade de Sao Paulo Faculdade de Medicina, BRAZIL

## Abstract

The World Health Organization estimates a 25% increase in anxiety and depression prevalence during the COVID-19 pandemic. 50% of surveyed US healthcare workers reported increased anxiety, and 27% of Chinese health professional students reported psychological distress. The mental health of US health professional students and their coping mechanisms, especially during an adverse time such as the pandemic, is less well understood. This study examined the US health professional students’ lifestyle behaviors and their association with the prevalence of poor mental health days. 890 students across seven health sciences colleges in 2020 and/or 2021 were recruited using convenience sampling. Participants completed socio-demographic questions and items from the U.S. Centers for Disease Control and Prevention’s (CDC) Behavioral Risk Factor and Surveillance System (BRFSS) 2019 survey. The participants reported a median of 7 days with poor mental health (IQR: 3-15 days) in the past 30 days. Female sex (OR 1.70, 95% CI [1.21, 2.38]), Asian race (OR 1.47, 95% CI [1.06, 2.06]), adverse childhood events (OR 2.01, 95% CI [1.45-2.78]), and frequent cannabis use (OR = 2.03, 95% CI [1.14-3.61]) were each associated with an increased risk of poor mental health during the COVID-19 pandemic. Exercise (OR 0.64, 95% CI [0.42-0.97]) was found to be a protective factor during COVID-19. These results indicate the need to design, implement, and evaluate mental health support services for health professional students, particularly among certain demographic groups. Students who are frequent cannabis users or have significant childhood trauma are more likely to have poor mental health and, as such, may benefit from additional support. A lifestyle psychiatry approach to overall wellness may offer students valuable and holistic coping mechanisms that incorporate lifestyle behaviors known to positively impact mental health.

## Background

The World Health Organization estimates a 25% increase in anxiety and depression prevalence during the COVID-19 pandemic [1]. Fifty percent of surveyed US healthcare workers [2] reported increased anxiety, while 27% of US-based health professional students who identify as Chinese reported psychological distress [3].The COVID-19 pandemic adversely affected the mental health of healthcare workers due to several occupational factors such as extended work hours, concerns about occupational exposures to the SARS-CoV-2 virus for self and family, and limited access to personal protective equipment (at the start of the pandemic) [2,4]. Health professional students, who were soon to join the workforce, also faced occupational limitations related to substantial disruptions in education, including a pivot from in-person to digital learning and training, virtual socialization, and uncertainty about the efficacy of their training [2,4–6]. Health professional students as a conglomerate population have not been extensively studied, especially within the United States (US), and few studies exist on this population, especially during the COVID-19 pandemic. Within nursing and medical student populations internationally, the pandemic was associated with higher rates of anxiety, depression, and distress [3,4,7–9]. There is a need for empirical information on mental health within the health professional student body, the precursors to the healthcare workforce, which faces a burnout epidemic [10].

This study assesses the association between various lifestyle behaviors or demographic factors (adverse childhood events, substance use (alcohol and cannabis), racial identity, diet, and exercise) and poor mental health days for health professional students across seven health sciences colleges (applied health, dentistry, medicine, nursing, pharmacy, public health, and social work) during the COVID-19 pandemic. Such information could help inform the need for support services that address mental health during the training of health professional students.

## Review of the literature related to mental health and lifestyle behaviors among health professional students

The studies on each variable are reviewed separately in the following sections.

### Adverse childhood experiences (ACEs)

Adverse childhood experiences (ACEs) impact both early development and long-term health outcomes well into adulthood [11,12]. A higher number of ACEs, more than 4 out of 10, are correlated with a clinical level of significance for the prevalence of mental health problems later in life [13,14]. However, it is important to note that ACEs have a dose-response relationship to serious health problems later in life. Even one ACE in a person’s life can be significant, and experiencing multiple ACEs could lead to a compounded effect [15]. Past research has established that young adults, such as health professional students, are more dependent on their social networks to cope with the effects of negative emotional experiences such as ACEs [16]. During the COVID-19 pandemic, social networks were threatened by lockdowns, job loss, limits on social gatherings, and limited opportunities to initiate and foster long-term, intimate relationships [16]. Alradhi and colleagues found that young adults in Canada with significant ACEs were more vulnerable to COVID-related mental health reductions [16]. Approximately 16% of the general population in the United States has experienced four or more ACEs [16–18]. Within the United States, health professional students have significantly higher rates of ACEs than the general population. Nursing and social work [19] students have a higher percentage of ACEs versus the national average with one study finding 23% of Bachelor’s of Nursing [20] students and another study finding 42% of Master’s of Social Work [21] students at a single institution were noted to have 4 or more Aces. Certain subspecialties of healthcare careers may attract people with different backgrounds and motivations, so different subspecialties may inherently have different prevalences of ACEs. For example, social workers may have chosen their careers due to an interest in understanding their own experiences, which spurred an interest in helping others do the same [22].

### Substance use

The substantial life changes and stressors of the COVID-19 pandemic led students and healthcare workers to resort to coping mechanisms such as alcohol consumption and cannabis use [23,24].

#### Alcohol use.

Individuals with pre-existing mental health issues are ten times more likely to engage in alcohol consumption [25]. Healthcare workers reported increased levels of alcohol abuse due to increased stress levels during the frontline pandemic response [26], which also led to increased mistakes on shifts [24]. In the US, Schepis et al. found that alcohol use was 34% more common after the announcement of the lockdown than before the pandemic [27]. Amongst nursing students in Israel, mental disengagement in the form of alcohol became more prominent near the end of the lockdown [7]. Alcohol consumption in this population group was associated with higher odds of moderate anxiety [7].

#### Cannabis use.

The relationship between cannabis use and mental health outcomes such as anxiety and depression has been investigated among undergraduate students, and Buckner et al. suggest an association between increased cannabis use and increased anxiety and depression [28]. A systematic review published in Oct 2018, prior to COVID-19, included studies from all over the world and found over 30% of medical students globally used cannabis, with a greater proportion of users being male [29]. Exposure to diverse stressors, burnout, and relatively easy access to drugs increase health professional students’ vulnerability to substance use. Substance use may affect students’ current academic performance and mental health [28–30]. Among similarly aged college students at other US universities, recent studies have found that cannabis use increased after the start of COVID-19, with an associated increase in depressive and anxiety symptoms [27,31]. Overall, substance use data on college students in the US provides valuable insights into the health professional student population nationwide, given many undergraduate students are enrolled in health profession programs like those in the applied health sciences or undergraduate nursing programs, but also provides context for students who may eventually enter graduate health professional programs as well.

### Racial identity

There is much evidence that the pandemic had harmful effects, specifically on racial and ethnic minorities [32]. The discovery that the COVID-19 outbreak originated in Wuhan, China fueled negative sentiment and actions against people of Asian complexions [33]. According to Tiwari and Zhang, Asian Americans already had higher rates of poor mental health pre-pandemic that negative attitudes and actions may have exacerbated [32]. Asian-Americans who were not personally subjected to acts of hate and violence still experienced mental exhaustion from being surrounded by the news of such incidents, in addition to the overall stress and anxiety caused by the COVID-19 pandemic [33]. Among Asian medical and biomedical graduate students, rates of anxiety and depression increased during the pandemic compared to the years prior [9].

### Diet

The recommended guideline for fruit and vegetable intake for young people and adults is five portions per day [34]. A systematic review by Glabska et al. found that multiple studies agree that fruit and vegetable intake is associated with lower levels of psychological stress, improved well-being, enhanced happiness, and decreased depressive symptoms [34]. Dharmayani et al. concluded that the daily consumption of fruit by young people aged 18 to 29 years was a contributing factor to mental health stability [35]. They also concluded that the consumption of vegetables once a day among women revealed a lower risk of adult depression [35]. Disruption in education and financial burden during COVID-19 may have impacted health professional students’ diet. Among college students in the U.S. food insecurity increased by 59.6% [36]. Less is known about food insecurity and decreased intake of fruits and vegetables among health professional students.

### Exercise frequency and type

Physical activity is essential to improve and/or maintain physical and mental health and enhance quality of life [37]. Individuals who exercised had 43% fewer days of poor mental health in the past month than individuals who did not exercise [38]. This association was strongest for individuals who exercised between 30-60 min per session and a maximum of 90 min, 3 to 5 times per week [38]. During the pandemic, home-based physical activities often became the safest exercise method. Home-based physical activities such as yoga and dancing were shown to alleviate anxiety, mood, and emotional health for medical students [37]. Exergames, exercises based on video games, provided a social outlet for users to engage with friends while participating in team physical activities [37].

### Aims

This study describes the association between lifestyle behaviors and demographic factors on the mental health of health professional students during the COVID-19 pandemic using the Healthy Days measure from the validated U.S. Centers for Disease Control and Prevention’s (CDC) Behavioral Risk Factor and Surveillance System (BRFSS) 2019 survey (see Methods section ‘Mental Health’ for the specific question posed to participants). Their behaviors can inform best practices for mental healthcare interventions and curriculum building in academic institutions.

## Methods

### Study design

**Fig 1:**
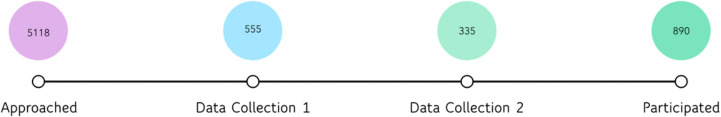
Recruitment Flowchart for HOLISTIC cohort study: Enrollment Trends from 2021 to 2022.

The Health Professional Students at the University of Illinois Chicago (HOLISTIC) Cohort Study is a prospective cohort study to assess the health of health professional students [39]. This study (# 2021-0114) was approved by the University of Illinois (UIC) IRB. Participants consented to participate with an e-consent, which was part of the software used to administer the survey. The current report examines baseline data in participants who enrolled in 2021 (n = 555) and 2022 (n = 355) (Fig 1). Recruitment occurred during the following periods: April 14, 2021 – May 5 2021 and March 30 2022 – June 1 2022.

### Study population

The HOLISTIC Cohort Study enrolled students across seven health science colleges (applied health sciences, dentistry, medicine, nursing, pharmacy, public health, and social work) at the University of Illinois Chicago, a U.S. Department of Education designated minority-serving institution [40]. Students were eligible to enroll in the HOLISTIC Cohort Study if they were age 18 years or older and enrolled full- or part-time in a health science program that prepares its graduates to enter a healthcare profession.

### Questionnaire: Behavioral risk factor surveillance system

Participants completed the CDC’s Behavioral Risk Factor Surveillance System (BRFSS) 2019 [41] survey online. The BRFSS is a comprehensive, national public health survey that collects data on health-related behaviors, chronic conditions, and use of preventive services among adults in the United States. It is the largest continuously conducted health survey in the world. In 2019, the BRFSS surveyed over 400,000 adults across all 50 states, the District of Columbia, and U.S. territories. The survey focuses on a wide range of topics, including: 1. Health behaviors: Smoking, alcohol consumption, physical activity, and nutrition; 2. Chronic conditions: Such as diabetes, heart disease, asthma, and arthritis; 3. Preventive health measures: Vaccinations, screenings, and health checkups; 4. Mental health: Including depression and psychological distress; 5. Access to healthcare: Health insurance coverage, use of healthcare services, and healthcare needs. The BRFSS is self-reported data, which is weighted to ensure it is representative of the adult population in each state. The survey helps the CDC and other health organizations track public health trends and inform policy decisions at the national, state, and local levels [41]. The health status domain includes items about Adverse Childhood Experiences (ACEs) [42], potentially traumatic events that occur in childhood (0-17 years). The mental health variable was assessed using the BRFSS self-reported question, “Now thinking about your mental health, which includes stress, depression, or difficulties with emotions, for how many days during the past 30 days was your mental health not good?”

### Statistical analysis

Mental health status was categorized into three groups based on the number of days with poor mental health in the past 30 days (at the time of survey response): good (less than 7 days), medium (7 to 13 days), and poor (more than 13 days) [43,44]. Lifestyle behaviors and demographic factors were further operationalized and summarized based on the original survey responses [Table 1]. Descriptive statistics were utilized to characterize mental health status, demographic variables, and lifestyle behaviors of the study population across seven health science programs. For continuous variables, the median and interquartile range (IQR) were presented, while for categorical variables, counts and percentages were presented. The multiple ordinal logistic regression was fitted to investigate the associations between multiple lifestyle behaviors and mental health. Multiple imputation was employed to address missing values while fitting the model. Additionally, a mixed graphical Gaussian model was fitted to visualize the conditional associations between the factors of interest. All statistical analyses were performed using R (version 4.1.3) software packages. A statistically significant association was defined as a two-sided p-value of less than 0.05, with a 95% confidence interval used for estimation.

**Table 1:** Operationalization of survey questions for lifestyle behaviors information.

Variable	Question	Operationalization
Number of Poor Mental Health Days[44]	Now thinking about your mental health, which includes stress, depression, or difficulties with emotions, for how many days during the past 30 days was your mental health not good?	a. Less than 7 days b. 7 days - 13 days c. 14+ days - Significant mental distress, anxiety, or depression
Adverse Childhood Experiences[13,14]	11 related questions in HOLISTIC’s Adverse Childhood Experiences Section	The number of Adverse Childhood Experiences (ACEs) for each student was calculated and then classified into two categories: those with 4 or more ACEs and those with fewer than 4 ACEs.
Alcohol Use[45]	One drink is equivalent to a 12-ounce beer, a 5-ounce glass of wine, or a drink with one shot of liquor. During the past 30 days, on the days when you drank, about how many drinks did you drink on the average?	a. Current light drinker (Men and Women 3 or less drinks) b. Current moderate drinker (Women: 4 to 7 drinks, Men: 4 to 14 drinks) c. Current heavy drinker (Women: more than 7, Men: more than 14)
Cannabis Use[46]	During the past 30 days, on how many occasions did you use marijuana or cannabis?	a. Not current user: 0 days b. Occasional user: 1-9 days c. Frequent user: more than 10 days
Diet[47,48]	6 related questions in HOLISTIC’s Fruits and Vegetables Section	Each occasion of intake of any of these types of fruits or vegetables was counted as 1 serving. The American Heart Association recommend that adults eat at least 4-5 serving of fruits and veggies per day. a. Below recommended veggies/fruit intake d. Recommended veggies/fruit intake
Exercise [49–51]	6 related questions in HOLISTIC’s Exercise Section	Exercise level of each student were identified by their frequent exercise type (light, moderate or vigorous), average exercise length per time and average exercise frequency per week. The recommend level of exercise is (i) with moderate physical activity at least 30 minutes per day on at least five days per week; (ii) with moderate physical activity at least 150 mins per week; or (iii) with vigorous physical activity at least 20 minutes per day on at least three days per week. a. Level I: no physical activity b. Level II: below recommended level c. Level III: recommended level

## Results

### Participant characteristics

A total of 890 participants were included in the analyses in the current report (555 participants enrolled in 2021 and 335 participants enrolled in 2022) [Table 2]. Participants were enrolled from the following colleges: the College of Medicine (n = 234, 26.3%); College of Nursing (n = 159, 17.9%), College of Pharmacy (n = 146, 16.4%), College of Applied Health Sciences (n = 123, 13.8%), School of Public Health (n = 78, 8.8%), College of Dentistry (n = 75, 8.4%), and Jane Addams College of Social Work (n = 74, 8.3%) [Table 2]. The median age of the participants was 25 years, with 79.0% identifying as female at birth and 53.6% identifying as non-white [Table 2].

**Table 2:** Participants’ demographical information of study population across seven health science colleges.

		Overall	COM	CON	COP	COAHS	SOPH	COD	JACOSW
**n**		890	234	159	146	123	78	75	74
**Age (median [IQR])**		25.00 [23.00, 28.00]	26.00 [24.00, 27.00]	26.00 [23.00, 31.00]	25.00 [23.00, 26.00]	24.00 [23.00, 26.00]	25.00 [24.00, 28.00]	26.00 [24.00, 30.00]	27.00 [24.00, 30.00]
**Sex at birth (%)**	**Male**	186 (21.0)	77 (33.2)	9 (5.7)	45 (31.0)	22 (17.9)	10 (12.8)	20 (26.7)	3 (4.1)
	**Female**	700 (79.0)	155 (66.8)	149 (94.3)	100 (69.0)	101 (82.1)	68 (87.2)	55 (73.3)	71 (95.9)
**Race (%)**	**White, Not Hispanic**	399 (46.4)	95 (42.0)	90 (57.3)	45 (33.1)	68 (55.7)	41 (53.9)	20 (29.0)	40 (55.6)
	**Asian, Not Hispanic**	220 (25.6)	62 (27.4)	28 (17.8)	59 (43.4)	28 (23.0)	12 (15.8)	27 (39.1)	4 (5.6)
	**African American, Not Hispanic**	42 (4.9)	12 (5.3)	6 (3.8)	7 (5.1)	2 (1.6)	3 (3.9)	7 (10.1)	5 (6.9)
	**Hispanic**	124 (14.4)	31 (13.7)	26 (16.6)	10 (7.4)	15 (12.3)	18 (23.7)	6 (8.7)	17 (23.6)
	**Other, Not Hispanic**	74 (8.6)	26 (11.5)	7 (4.5)	15 (11.0)	9 (7.4)	2 (2.6)	9 (13.0)	6 (8.3)

Abbreviations: College of Medicine (COM), College of Nursing (CON), College of Pharmacy (COP), College of Applied Health Sciences (COAHS), School of Public Health (SOPH), College of Dentistry (COD), Jane Addams College of Social Work (JACOSW)

### Mental health

Participants reported a median of 7 days with poor mental health (IQR: 3-15 days) in the past 30 days, with 48.6% indicating good mental health, 23.6% medium mental health, and 27.8% poor mental health [Table 3].

**Table 3:** Participants’ mental health lifestyle behaviors across seven health science colleges.

		Overall	COM	CON	COP	COAHS	SOPH	COD	JACOSW
**Poor mental health days (past 30 days) (median [IQR])**		7.00 [3.00, 15.00]	5.00 [2.00, 13.50]	8.00 [5.00, 12.00]	7.00 [3.00, 15.00]	7.00 [4.00, 15.00]	10.00 [5.00, 17.25]	5.00 [1.50, 10.00]	10.00 [5.00, 15.00]
**Poor mental health days**	Less than 7 days	431 (48.5)	132 (56.4)	71 (44.7)	67 (46.2)	60 (48.8)	32 (41.0)	43 (57.3)	26 (35.1)
	7 days - 13 days	210 (23.6)	43 (18.4)	50 (31.4)	30 (20.7)	27 (22.0)	19 (24.4)	16 (21.3)	24 (32.4)
	14+ days	248 (27.9)	59 (25.2)	38 (23.9)	48 (33.1)	36 (29.3)	27 (34.6)	16 (21.3)	24 (32.4)
**Adverse** **Childhood** **Experiences (%)**	less than 4	710 (79.8)	198 (84.6)	119 (74.8)	121 (82.9)	106 (86.2)	56 (71.8)	68 (90.7)	42 (56.8)
	4 or more	180 (20.2)	36 (15.4)	40 (25.2)	25 (17.1)	17 (13.8)	22 (28.2)	7 (9.3)	32 (43.2)
**Alcohol Use (%)**	Current light drinker	541 (63.9)	143 (62.4)	97 (64.7)	98 (73.7)	73 (61.3)	47 (60.3)	46 (70.8)	37 (51.4)
	Current moderate drinker	225 (26.6)	72 (31.4)	34 (22.7)	26 (19.5)	34 (28.6)	21 (26.9)	14 (21.5)	23 (31.9)
	Current heavy drinker	81 (9.6)	14 (6.1)	19 (12.7)	9 (6.8)	12 (10.1)	10 (12.8)	5 (7.7)	12 (16.7)
**Cannabis Use (%)**	Not current user	702 (81.4)	191 (85.3)	133 (85.3)	131 (93.6)	96 (79.3)	45 (58.4)	62 (88.6)	44 (60.3)
	Occasional user	107 (12.4)	30 (13.4)	17 (10.9)	5 (3.6)	17 (14.0)	17 (22.1)	7 (10.0)	13 (17.8)
	Frequent user	53 (6.1)	3 (1.3)	6 (3.8)	4 (2.9)	8 (6.6)	15 (19.5)	1 (1.4)	16 (21.9)
**Diet (%)**	Below recommended vegetable/fruit intake	593 (72.0)	166 (74.4)	101 (68.7)	102 (82.9)	77 (66.4)	47 (62.7)	47 (69.1)	52 (73.2)
	Recommended veggie/fruit intake	231 (28.0)	57 (25.6)	46 (31.3)	21 (17.1)	39 (33.6)	28 (37.3)	21 (30.9)	19 (26.8)
**Exercise level (%)**	level 1	139 (16.3)	20 (8.9)	24 (15.5)	52 (38.8)	9 (7.4)	9 (11.8)	16 (24.2)	9 (12.3)
	level 2	461 (54.1)	125 (55.6)	94 (60.6)	46 (34.3)	71 (58.2)	42 (55.3)	33 (50.0)	50 (68.5)
	level 3	252 (29.6)	80 (35.6)	37 (23.9)	36 (26.9)	42 (34.4)	25 (32.9)	17 (25.8)	14 (19.2)

Abbreviations: College of Medicine (COM), College of Nursing (CON), College of Pharmacy (COP), College of Applied Health Sciences (COAHS), School of Public Health (SOPH), College of Dentistry (COD), Jane Addams College of Social Work (JACOSW)

### Demographic factors and lifestyle behaviors associated with mental health

**Fig 2:**
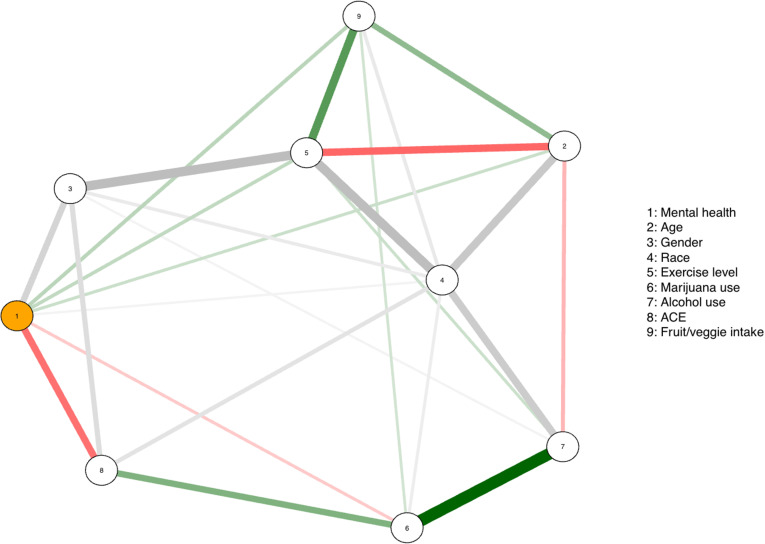
Network visualization of association between variables of interest. The edges between two nodes indicate the presence of an association between two factors. The width of the edge represents the strength of the association, with thicker edges indicating stronger associations. Red indicates a negative association, while green indicates a positive association. Grey is used when there are one or more nominal variables involved.

The ordinal logistic regression analysis identified significant relationships between mental health and several demographic variables, such as age, assigned sex at birth, and race, with an odds ratio (OR) exceeding 1, indicating a higher chance of poor mental health days. Self-identified Asian (OR 1.47, 95% CI [1.06, 2.06]) and female students (OR 1.70, 95% CI [1.21, 2.38]) had higher odds of poor mental health, while younger students (OR 0.97, 95% CI [0.94, 0.99]) were more likely to report good mental health [Table 4]. Notably, more than 4 ACEs were strongly associated with mental health (OR 2.01, 95% CI [1.45-2.78]) [Table 4]. Cannabis use (OR = 2.03, 95% CI [1.14-3.61]) and exercise (OR 0.64, 95% CI [0.42-0.97]) were found to be significantly associated with mental health [Table 4]. There were no significant associations between poor mental health days and the lifestyle behaviors of alcohol use and intake of fruits and vegetables [Table 4]. Furthermore, the network visualization presented in Fig 2 illustrates complex associations between various demographic and lifestyle variables.

**Table 4:** Multiple ordinal logistic regression analysis.

Covariate		Odds Ratio (95% Confidence Interval)	P value
**Age**		0.966 (0.940, 0.993)	0.014
**Sex at birth**			
	Male	Reference	Reference
	Female	1.697 (1.210, 2.379)	0.002
**Race**			
	White, not Hispanic	Reference	Reference
	Asian, not Hispanic	1.474 (1.057, 2.056)	0.023
	African American, not Hispanic	0.862 (0.467, 1.593)	0.636
	Hispanic	1.038 (0.705, 1.530)	0.849
	Other, not Hispanic	0.998 (0.608, 1.639)	0.994
**ACE**			
	less than 4	Reference	Reference
	4 or more	2.011 (1.454, 2.782)	<0.001
**Alcohol**			
	Current Light drinker	Reference	Reference
	Current moderate drinker	0.921 (0.670, 1.267)	0.615
	Current heavy drinker	1.119 (0.711, 1.762)	0.627
**Cannabis Use**			
	Not current user	Reference	Reference
	Occasional user	1.219 (0.815, 1.823)	0.335
	Frequent user	2.028 (1.139, 3.610)	0.017
**Diet (vegetable and fruit intake)**	Below recommended veggie/fruit intake	Reference	Reference
	Recommended veggie/fruit intake	0.755 (0.559, 1.019)	0.067
**Exercise**			
	Level I	Reference	Reference
	Level II	0.759 (0.525, 1.097)	0.142
	Level III	0.643 (0.424, 0.975)	0.038

Female sex, Asian Race, 4 or more ACEs, and frequent cannabis use are positively associated with poor mental health. Level III exercise (students who exercise more than the recommended amount) is negatively associated with poor mental health.

## Discussion

We assessed health professional students’ lifestyle behaviors and mental health during the COVID-19 pandemic. In our sample, most students endorsed at least a week of poor mental health days in a month’s time. Older students had a higher prevalence of poor mental health days. Students who identified as female and/or Asian had a higher prevalence of poor mental health days in a month. Students who heavily used cannabis or experienced 4 or more ACEs were also more likely to experience an increased number of poor mental health days during the pandemic. Students who exercised experienced fewer poor mental health days, likely indicating less psychological distress.

Age was a significant factor, older students in our cohort had a higher prevalence of poor mental health days. It should be noted that the age range of our cohort was 23 to 30. While this is a narrow range, it reflect the makeup of the health professional student body. It could be hypothesized that older student may experience increased academic and financial pressures, more significant life stressors, or challenges related to balancing responsibilities like work, family, and studies. These added pressures may contribute to higher levels of stress, anxiety, and other form of mental health distress.

Regarding childhood trauma, we found 20.2% of students identified as having a significant number of ACEs, defined as 4 or more ACEs. This is slightly higher than the national average of 16%, which is consistent with comparable studies [52]. Social work, public health, and nursing students had a 43.2%, 28.2%, and 25.2% higher likelihood of significant ACEs compared to our general student population, respectively [17,21]. In our sample, students with significant ACEs were almost twice (2.01) as likely to have worse mental health in comparison to all participants. Given the increased prevalence of ACEs among social work students and direct training exposure to patients undergoing ACEs, this group may require additional health promotion and therapy resources as their work may trigger emotions from their past. ACEs have been associated with emotional dysregulation and low coping skills. Added stressors from the pandemic and exposure to patient suffering during their education may explain the worse mental health of health professional students with significant ACEs [17].

This study found no significant association between light, moderate, or heavy drinkers and their number of poor mental health days. A previous study on Polish medical students found that alcohol consumption changes depending on the scenario [23]. During the lockdown, social drinking was no longer a common option. As such, the lack of significant association between drinking and poor mental health in our study may be due to the change in circumstances associated with alcohol consumption during the pandemic. Our survey did not include questions about the social situations surrounding drinking.

When examining cannabis use among health professional students, we found an association between the frequency of cannabis use and mental health. Health professional students who were frequent cannabis users had twice the odds of poor mental health compared to those who had never used cannabis. Previous studies of undergraduates found a similar association between frequent cannabis use and poor mental health [27,31]. We continue to see this trend in health professional students, even though they may be more informed about the risks. Social cannabis use has been shown to increase cannabis use frequency [53,54]. However, research to date that has compared the relative risk profiles of social and solitary cannabis users suggests that using alone is riskier than using with others [55]. Increasing education, training, and public service announcements to inform health professional students of the updated risk data for solitary cannabis use may help decrease cannabis use frequency. Primary care providers should consider including screening about cannabis use during substance use and mental health conversations with health professional students due to the association between these factors and the increasing prevalence of cannabis use. Further research is still necessary to establish a causal relationship between cannabis use and mental health.

Within our study, only Asian identity was associated with a significantly higher number of poor mental health days compared to other races. Our study demonstrated that Asian students had a 47% increase in the odds of poor mental health compared to white students (p<0.05) [Table 4]. Other race categories were also compared; however, there was no statistically significant difference [Table 4]. Tiwari and Zhang noted a sevenfold increase in the prevalence of depression and anxiety symptoms amongst Asian Americans during the pandemic [32]. Experiencing or witnessing negative sentiment towards Asian Americans due to the pandemic could have further exacerbated the pandemic’s already negative impact on mental health for Asian students [33]. In times when groups are targeted by negative attitudes and sentiments, university officials should be prompt in providing additional mental health resources to target groups.

An overwhelming majority of our participants (72%) reported consuming less than the recommended five portions of fruits and vegetables daily. Our findings demonstrated marginal significance (p = 0.07) between fruit and vegetable intake and mental health [Table 3], which reflects the conflicting conclusions present in the literature [34,35]. Students who consumed more than the recommended five portions of fruits and vegetables daily reported having 75% fewer poor mental health days per month (i.e., less than seven days out of the month). However, students consuming the recommended number of fruits and vegetables still experienced more than seven poor mental health days per month. It should be noted that this study did not include a variable for financial trouble or food insecurity, which could constitute a standalone cause of poor mental health [56]. Therefore, the effect of not eating the recommended portions of fruits and vegetables on mental health cannot be clearly established. Future versions of this study will need to consider a food insecurity variable to determine whether it impacts health professional students’ mental health in relation to their diet.

Lastly, students who exercise more than the recommended level were 46% less likely to experience poor mental health than students who do not exercise. Any amount and type of physical activity has been shown to have mental health benefits, though no particular type was more significantly beneficial than others in previous studies [37]. In our study, the greatest decrease in poor mental health days was found to be associated with moderate exercise types, which included bicycling, yoga, and dancing, aligning with previous studies [37]. 83.7% of health professional students engage in moderate exercise [Table 2]. As such, institutions providing intramural sports and moderate/vigorous exercise programs in convenient locations for health professions students is important to continue these beneficial health habits.

Lifestyle psychiatry is an innovative approach to physical and mental health treatment plans that integrates pharmacologic and lifestyle interventions to build effective and holistic treatment plans [57]. The challenge with lifestyle interventions is that the change must be sustained to perceive a sustained benefit [57]. Therefore, patients often find it easier and quicker to opt for pharmacologic interventions. Psychiatrists have the particular advantage of expertise in motivation, effective behavior change strategies, and relapse management, all necessary factors to form healthier habits [57]. Given that the previously mentioned lifestyle experiences and behaviors have a strong association with mental health in this group, universities could consider consulting lifestyle psychiatrists when creating wellness programming. Healthcare professional students are a unique subset of the general population because they not only encounter regular student stressors and financial difficulties due to high tuition or debt rates but also stressors like those of current healthcare workers, a group who is well-established to have high burnout levels [10] These students are held to similarly high societal professionalism expectations as those of healthcare workers. With increased workloads as they mature into their roles as healthcare professionals, these students are likely to have further exacerbated levels of stress and burnout. Providing instruction on how to utilize lifestyle psychiatry as part of their wellness programming will help these soon-to-be doctors, nurses, pharmacists, etc. build stronger foundations for burnout prevention as they adopt roles requiring optimized cognitive performance with little to no margin for error.

## Limitations

The main limitation of this study is that a single, self-reported measure, the BRFSS Healthy Days, was used to assess mental health, which is a multifactorial issue. Although the BRFSS is widely used and accepted as a simple quantitative measure of mental health, it is difficult to ascertain which aspects of mental health the respondents are considering when they answer. This measure is instead best used to assess the interference on an individual’s life due to poor mental health. Future studies could use a clinical measure such as the Patient Health Questionnaire (PHQ-9) for more specific data on poor mental health symptoms. To eliminate potential responder or recall bias associated with self-reporting for all measures, future studies can validate survey responses with electronic health records and governmental contact tracing databases in addition to self-reporting for lifestyle factors such as food insecurity, specific mental health diagnoses, COVID symptom presentation and vaccination status, patient exposure, and adherence to social distancing measures.

Further limitations of this study included the lack of precision and specificity in assessing the quantity of substance use and intake of fruits and vegetables. Study designs with measures that specify serving sizes for substances (alcohol, cannabis) and fruits/vegetables could address this limitation. Our data was collected with study participants from a single, Midwestern minority-serving public university; it is not known if findings from the HOLISTIC Cohort Study would apply to health professional students elsewhere. Multi-center studies that employ the protocol[39] of this study across multiple public and private universities in the U.S. and internationally could be used to address the potential for limited generalizability from the HOLISTIC Cohort Study.

## Conclusion

The HOLISTIC Cohort Study has established a platform for assessing the health of health professional students educated during a unique time in history –– the COVID-19 pandemic. To our knowledge, no other cohort study has evaluated a comprehensive set of lifestyle behaviors, mental health status, and occupational trajectories as students trained during the COVID-19 pandemic enter the health professional workforce. Health professional students’ mental health status during COVID-19 can be negatively correlated with female sex and racial Asian identity. This follows the well-documented trend that depression, especially stress-induced, is more prevalent in females than in males [58]. Asian students experienced especially increased stress levels during the COVID-19 pandemic due to the negative sentiments and actions they experienced or witnessed. Students who were frequent cannabis users or had significant childhood trauma were more susceptible to poor mental health and as such would benefit from additional mental health support. Despite decreased access to gyms during lockdown, home-based (yoga) or singular exercise (biking) was a protective health behavior in our study population. Health promotions around home-based exercise and intramural sports to health professional students, along with longer open hours for the gym, may be beneficial. Consulting lifestyle psychiatrists to help build wellness plans may help universities tackle the prevalence of poor mental health in health professional students.

## Supporting information

S1 Data Raw data file. This file provides all of the raw data used for this study.
(CSV)
